# The management of COVID-19 in prisons – the case of Northern Ireland

**DOI:** 10.1186/s40352-025-00350-8

**Published:** 2025-07-01

**Authors:** Dermot O’Reilly, Janine Cooper, Rosie Murphy, Aideen Maguire, Richard Kirk, Trish Kelly, Ruth Gray, Stephen McGarrigle, Julie Anderson, Michael Donnelly

**Affiliations:** 1 Queen’s University Belfast, Belfast, UK; 2 South Eastern Health and Social Care Trust, Dundonald, UK; 3 Northern Ireland Prison Service, Belfast, UK

**Keywords:** Infection control, COVID-19, Coronavirus, Prison, Northern Ireland

## Abstract

**Background:**

This paper describes COVID-19 cases in the Northern Ireland (NI) prison population during the pandemic in relation to the general population and changes implemented to control infection in NI prisons. Data obtained from the Department of Justice and Department of Health (week ending 22nd May 2020 to week ending 8th April 2022) are presented using descriptive statistics. An account based on information about meetings, activities, routines and processes in prison during the pandemic was gathered mainly via an interview with a Healthcare in Prison staff member. This narrative was refined following feedback from the lead Northern Ireland Prison Service (NIPS) liaison person to provide an overview of infection control measures, adaptations and cases.

**Results:**

Strict infection prevention and control measures introduced in NI prisons, such as screening on entry and isolation periods, restricted access, halting all non-essential activities and providing additional wash stations were successful in minimising the onset and spread of COVID-19. The integrated NIPS-HiP approach appeared to prevent COVID-19 infections for most of the pandemic, and the waves of peak infection that characterised spread in the general population were not evident in the prison population. This management approach in prisons was characterised by multiagency partnership involving the NI Public Health Agency, joined-up planning and collaborative working.

**Conclusions:**

This study describes the implementation of infection control measures in NI prisons during the COVID-19 pandemic and contributes to our understanding/planning about the prevention and management of similar scenarios in the future.

**Supplementary Information:**

The online version contains supplementary material available at 10.1186/s40352-025-00350-8.

## Background

The potential for prisons to concentrate and spread disease led them to be dubbed ‘incubators of infectious diseases’ (Howard, 1973). This view dates back to the 16th century when typhus, known as ‘gaol fever’, was responsible for high mortality in prisons (Howard, 1973]). Prisons may act also as vectors for community transmission. For example, the early release of people in prison in Russia to ease overcrowding, many of whom had tuberculosis (TB), contributed to a global resurgence of TB disease (Coker, 2001; Mercer et al., 2003]. Thus, health in prisons is an integral part of public health, generally (Cloud et al., 2023), and, in particular, with respect to the control of infectious diseases such as the coronavirus disease 2019 (COVID-19) pandemic (Kinner et al., 2020; Simpson & Butler, 2023).

The prison population is at increased risk of infectious disease due to an array of individual and institutional factors. This population comprises a disproportionate number of people from disadvantaged backgrounds with low levels of educational attainment, and a greater prevalence of chronic pre-existing medical conditions including poor mental health, who are more likely to indulge in risky or harmful behaviours such as smoking and intravenous and other drug use (Kinner & Young, 2018). In many cases, incarceration may be protracted and repeated committals may increase the risk of the spread of infection. People are often housed in environments with poor ventilation, which are conducive to the spread of infectious and contagious conditions. Overcrowding, though difficult to measure is a particular concern, and a recent review concluded that overcrowding was a major factor in the transmission of many infectious and communicable diseases in prisons (Simpson et al., 2019), as well as increasing the likelihood of misconduct and post-release recidivism (Gaes, 1985; Adamson et al., 2022).

During the COVID-19 pandemic, key challenges in daily prison operations included staffing issues, the implementation of public health policy, and maintaining correctional programs in the prison environment (Novisky et al., 2023). A review of peer-reviewed studies and grey literature relating to guidance about how to prevent and control COVID-19 in custody identified 201 publications and 374 different recommendations over 19 domains (Pearce et al., 2021). Studies that investigated the COVID-19 pandemic in prison highlighted the complexity of the setting in relation to implementing effective responses and reported that the screening of the prison population and prison staff, and the reduction of overcrowding, were the most common methods of COVID-19 prevention (Esposito et al., 2022). However, COVID-19 testing varied within and between countries, for example, in the USA and Canada, testing of the prison population was more frequent than the general population, but testing levels and coverage were dependent on the location (state/province) of a prison (Williams et al., 2024). Another recent review found that most studies about the delivery of healthcare services in prisons in high-income countries during the COVID-19 pandemic focused on drug treatment services and mental health services - examples of service delivery changes included dispensing processes, physical distance measures and telemedicine (Hearty et al., 2023). The COVID-19 pandemic alongside operational changes to reduce the risk of infection transmission such as social distancing and isolation, withdrawal of prison visits and reduced mental health services impacted negatively on the mental health status of prison populations (Johnson et al., 2021).

This paper presents the results from a case study of the management of the COVID-19 pandemic in the Northern Ireland Prison Service (NIPS) in terms of (i) the number and pattern of COVID-19 infections amongst the prison population in Northern Ireland (NI) during the pandemic; (ii) a comparison of prison and community infection; and (iii) the planning, preparation implementation and management of in-prison changes and their relationship to controlling infection.

## Methods

### Setting

The NIPS consists of a high security male prison; a medium-to-low security male prison; and a third site that houses young adults (i.e. committed from their 18th birthday to the day before their 21st birthday; if a period on remand or sentence starts before an individual is 21 years old and passes their 21st birthday, they may remain on this site until they are aged 24 years), and a separate prison for females. The NI South-Eastern Health and Social Care Trust (SEHSCT) manages the NI Healthcare in Prison (HiP) service and is responsible for the delivery of health and social care in the various prison settings. At the beginning of the COVID-19 pandemic, a plan was initially developed between NIPS and HiP to control the risk of infection transmission. Subsequently the local Public Health Agency (which has an overarching remit for protecting the public health of all citizens in NI) became involved, in collaboration with NIPS and HiP, to put its guidance in place (based on the Public Health England document at the time). This plan remained in draft form. NIPS developed its own Pandemic Plan and Procedures which went through 11 different iterations reflecting the different waves/phases of the pandemic. Infection control measures were informed by SEHSCT infection control advice, but it also included very strict instructions for NIPS staff/household, testing, isolation and contact tracing arrangements to stop infection coming into establishments. The plan was based on best guidance from various sources, mainly from the World Health Organisation (WHO) (WHO, 2020; WHO, 2021; Pearce et al., 2021).

### Key quantitative indicators for this study

It was anticipated that a successful pandemic response would be evidenced by infection amongst the NI prison population that was lower than the local NI community, including avoiding the ‘waves’ that were observed in the general population.

The study used the Weekly Situation Reports for the prison population in NI that were routinely published by the Department of Justice (DoJ, 2022). These reports detailed prison population by establishment and weekly committals, discharges and transfers as well as the cumulative number of confirmed COVID-19 cases per week. These data were available for the period of week ending 22nd May 2020 to week ending 8th April 2022 (inclusive). Daily dashboard updates about COVID-19 from the NI Government Department of Health (DoH) (DOH, 2024) reported comparable data for COVID-19 confirmed cases among the general population, covering the same period i.e. cases were collated and analysed weekly from week ending 22nd May 2020 to week ending 8th April 2022.

These data were examined to analyse changes in the prison population, percentage prison capacity in NI and the cumulative number of cases among the prison population over the study period. The proportion of each population subgroup (prison and general population) with new confirmed COVID-19 infections were key indicators. For the general population, the proportion was calculated using mid-year population estimates (NI general population on Census Day 21st March 2021 (*n* = 1,903,175) [NISRA, 2024]). For the prison population, the proportion affected was calculated using the population at the mid-point of the study period: the prison population in the week ending 26th March 2021 (*n* = 1,405) (DoJ, 2022).

An account of prison COVID infection control procedures.

An interview was conducted with a lead member of prison health staff with responsibility for COVID-19 management and we drew upon a separate but interrelated qualitative study in our prison conducted by lead co-author (Gray et al., 2021). This account of infection control measures, adaptations and cases was refined following feedback from the lead Northern Ireland Prison Service (NIPS) liaison person.

The case study of NI’s prisons provides insight into the policies and measures that were introduced during the pandemic. Qualitative studies in prison provide important insights into lived experiences during pandemic-like situations; and the results of a thematic analysis of the experiences of people incarcerated in NI’s prisons increased our understanding about the impact of isolation during COVID-19 and, in turn, enabled improvements to be made in the delivery of prison services (Gray et al., 2021). The HIP service-NIPS-PHA collaborative approach involved gathering data regularly about the changes to management routines and processes at the various prisons, including qualitative data about how the NI HIP service responded to the COVID-19 pandemic.

## Results

**Fig. 1:**
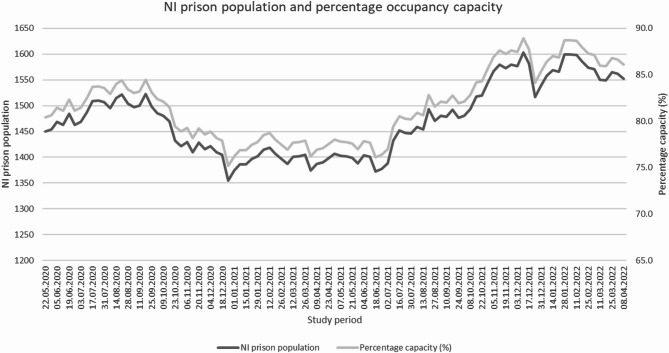
NI prison population during the study period and percentage occupancy capacity

Figure 1 shows the prison population at intervals during the study period and the associated occupancy capacity. The current approved operational capacity in NI’s prisons is for 1803 individuals [WHO, 2022]. Some individuals were given temporary early release at the beginning of the pandemic and during the study data collection period, subject to applied conditions (DoJ, 2020). This most likely explains why numbers and occupancy in May 2020 were lower than usual. Occupancy continued to fall during the latter part of 2020 and did not return to expected levels until autumn 2021. Occupancy was around 77% during most of this time which concurs with official statistics that report a 15.4% drop in admissions/receptions (WHO, 2022). Occupancy levels continued to increase reaching 88.7% by end 2021 and early 2022.

**Fig. 2:**
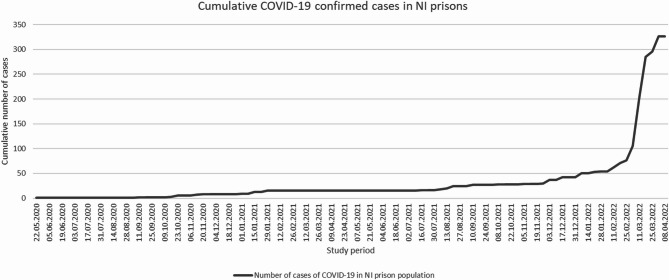
Cumulative COVID-19 cases in NI prison population

**Fig. 3:**
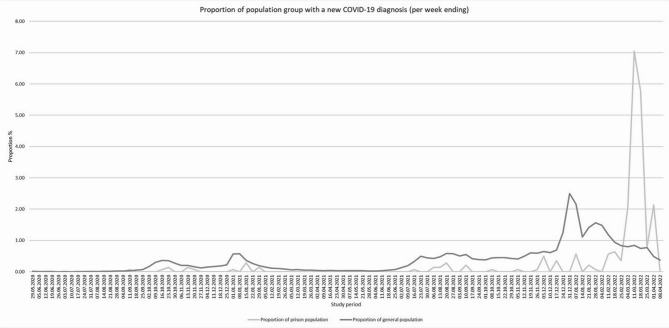
Proportion of prison and general population with a new COVID-19 infection diagnosis during the study period

The cumulative number of cases in NI prisons is shown in Fig. 2. A sudden large increase in cases occurred at the start of February 2022. Figure 3 shows the proportions of the prison and general population with a new confirmed COVID-19 infection throughout the study period. The familiar peaks of infections in the general population are characteristic of the waves seen throughout the NI population and elsewhere in the United Kingdom. However, no such waves were evident in the NI prison population, except for an increase in infections in February 2022. There were no COVID-19 deaths and no hospitalisations of people in prison during the study period. The weekly number of new COVID-19 confirmed cases among the Northern Ireland population during the study period with information on national lockdowns and public restrictions is shown in Appendix 1.

### Curtailments and mitigations

At the beginning of the pandemic (March 2020), NI prisons were free of COVID-19 and the underlying principles for the HIP service and NIPS COVID-19 response were to eliminate community-to-prison spread, to reduce the potential for intra-prison spread, and where possible, to mitigate the negative impact of associated increased restrictions of movement, visits and activities on prisoner wellbeing.

The NIPS, as with many other prisons across the world, operated under the precautionary principle and assumption that all new people in prison on entering the system were positive for COVID-19 until proven otherwise. All individuals were therefore screened on entry and, irrespective of symptoms, were required to spend 14 days in isolation and show a series of negative COVID-19 tests before entering the general prison community. This was possible because the NIPS was able to repurpose some existing but unused accommodation to allow for universal single-cell occupancy across prison settings. Additional measures to stop the infection entering the prison included halting face-to-face visits and restricting access to only essential workers defined as prison staff and healthcare workers. NIPS had robust procedures in place, which included PCR testing for staff through the Belfast and Northern Trusts (and the households of staff) in order to facilitate staff to isolate when they, (or household members) were symptomatic or tested positive, as well as robust contact tracing arrangements within the prison environment for staff and prison population who tested positive for COVID-19.

NIPS operated a landing-based regime or arrangement, and the prison population were not locked 23 h per day as applied in England and Wales (with the exception of those in isolation). Prison population movement and contact within the prison was reduced to a minimum and all non-essential activities, such as educational classes, church and gym were halted. As the pandemic progressed, virtual lessons were introduced, and other methods of church service and gym/exercise were provided. The importance of maintaining hand hygiene was emphasised and additional wash stations were made available throughout the prison. Some of the usual infection control measures advocated for the general population were not suitable for prison environment. The use of alcohol-based hand gel was prohibited due to the high risk of ingestion and abuse Doyon & Welsh, 2007), and the use of face coverings by people in prison was not used due to the threat of security risk and potential ligature risk. Clinically vulnerable people in prison were identified from their medical records and housed together under shielding arrangements. People in prison who developed a temperature, or other potential signs of infection were placed in isolation until their negative status could be confirmed.

Vaccination commenced in NI prisons at the same time as in the community (December 2020). Prison health staff and clinically vulnerable people in prison were vaccinated as a priority as they would have been in the general population. However, as per the DoH in Northern Ireland COVID-19 vaccine programme, neither the general prisoner population nor prison officers were given priority status for vaccination. It was noted that, overall, it was reasonable to expect that there would be a higher degree of vaccination hesitation amongst a prison population given the relationship between vaccine hesitancy and lower socioeconomic background. This tendency was further compounded by language difficulties, an inclination to believe in misinformation about the vaccination and the transitory nature of the population making completion of vaccination course difficult. However, a dedicated education campaign by the HIP service helped to mitigate the effects of these issues. People who received their first dose of vaccination and were released often returned to custody without receiving their second or booster doses. Prison was a significant setting to opportunistically increase vaccination rates amongst this cohort of the population. A WHO report used daily joint reporting from NIPS and HiP to provide a profile of NI regarding prison occupancy, cases and vaccination rates (WHO, 2022).

The NIPS initiated a process whereby, via virtual visits, the prison population could communicate with family members from 9th April 2020 and attend health care visits remotely. The isolation facilities for people in prison were improved in response to the results from a bespoke questionnaire - distraction packs, including word searches and competitions, were developed and made available by NIPS staff and HIP Engagement Teams. Entry into custody was recognised as a time when people may be feeling more vulnerable. In response to the new committal regime of 14 days isolation, a series of mitigation measures were implemented. The HiP Engagement Team conducted interviews weekly with a convenience sample of people who had completed committal isolation. This information was analysed, and weekly meetings were established with prison governors to make improvements to the isolation conditions and practices. These adaptations included improving access to healthcare appointments, sleep hygiene advice and a new mental health assessment pathway (Gray et al., 2021). Committal/symptomatic isolation was reduced to 10 days in December 2020, in line with DoH advice, and this was also revised in January and February 2022 in line with DoH guidance.

## Discussion

It was anticipated at the start of the pandemic that the prison population were at increased risk of becoming infected and suffering the consequences of COVID-19, and in many cases these predictions have been well founded (WHO, 2022). A series of papers have subsequently confirmed the higher rates of both infection and deaths from COVID-19 amongst people in prison than the general population globally (Gray et al., 2021; GOV.UK, 2021; Byrne et al., 2020). However, as this report shows, this was not the case in NI. The changes implemented by the NIPS/HIP service were successful in limiting the introduction and spread of COVID-19 in the NI prison system. The overall proportion of people infected in NI prisons was low compared to that of the general population, and the waves of peak infection that characterised spread in the general population were not evident in the prison population. COVID-19 infections in NI prisons in 2020/21 occurred in the isolation areas, not in the general prison population with the exception of some cases in October 2020. However, it was difficult to accurately estimate the infection in prisons as the population at risk varies over time. The use of mid-pandemic estimates for the prison population in the current study while introducing a degree of uniformity is an underestimate of the populations-at-risk especially for people in prison given the turnover, even during the pandemic. This may have resulted in an underestimation of the denominator. Prison population statistics (available from NIPS) were not used in this study, and this is a noted limitation in the analyses. Furthermore, statistical adjustments for the characteristics of the prison population and intrinsic risk of COVID-19 infection and associated morbidities could not be made due to limitations in the demographic data available.

Whilst it is difficult to be certain which aspect of the many reforms that were introduced over the course of the pandemic were most important in preventing widespread COVID-19 infection in the NI prisons, the collaboration, largely between NIPS/HiP, was a key significant contributing factor to the successful outcome for people in prison during this period. A joint group was formed and funded by the HSCB in June 2020 in response to a NIPS business case for ‘in-cell activity’. The collaborative group comprised the NIPS, HiP, HSCB, and PHA and examined collectively, among other operational aspects, activity regarding people in isolation areas. It is likely that many of the increased hygienic measures practiced in the wider community and implemented in the prison combined with increased social distancing and the screening, isolation and testing of suspected cases were effective in minimising risk and spread within the prison. The NIPS decision to minimise the flow of NIPS/healthcare staff into prison, along with very strict procedures for staff around household infection/symptoms and isolation, together with robust internal contact tracing were key factors in achieving the successful outcome. The early and safe release policies had been advocated as a way of reducing overcrowding and reducing the risk of transmission in prisons (Braithwaite et al., 2021; Davies & Keeble, 2021) was partially employed in NI (DoJ, 2020). However, it is clear that the preventive measures i.e. those processes aimed at stopping the infection getting into the prison in the first place were very effective. These were helped by the relatively low occupancy levels prior to the pandemic onset and the ability to quickly make additional space within the prison available for housing. Although the overall evidence of a causative relationship between overcrowding and risk of infectious disease is not strong (Simpson et al., 2019), US studies reported a 14% average increase in COVID-19 cases for every 10% increase in capacity filled, and that prisons exceeding 100% design capacity reported a 5-fold increase in COVID-19 incidence when compared to those below 70% design capacity (Simpson & Butler, 2020; UNODC, 2010). The enhanced capacity in NI prisons, which allowed for the isolation and quarantining of all new committals and of established population who were symptomatic, as well as increasing overall cell spatial density, required close collaboration between the management by NIPS and Healthcare in Prisons. This would not have been the case in many other prisons in the UK or world-wide. It is estimated that approximately 59% of all countries worldwide have prison occupancy levels exceeding their officially reported capacity and pre-pandemic levels in NI levels were 75.1% compared to 109.8% for England and Wales (Clemenzi-Allen & Pratt, 2021). Prison expansion, resulting in prison overcrowding making social distancing almost impossible was noted as a main reason for the higher COVID-19 infections and death rates in prisons in approximately 50 countries including the UK (Byrne et al., year cited in Gray et al., 2021).

While it is evident that the strict infection prevention and control measures that were introduced at the start of the pandemic were successful in minimising the spread and impact of COVID-19 in Northern Irish prisons, they also affected most aspects of prison life and the extent of the unintended consequences of these measures for people incarcerated in prison and prison staff is less clear and beyond the scope of the current study. As others have stated, it is unclear if the balance of, protection of, and burden on, the prison population has been achieved (Leibowitz et al., 2021). Others such as Suhomlinova et al. (2022) have reported on the lived experiences of people in prisons in England and Wales during the pandemic and detail the impact that the severe limitations of activities and human contact has had on well-being (HMIP, 2021; Suhomlinova et al., 2022; Luigi et al., 2020]. However, in NI individuals in the general prison population were not locked 23 h per day as they were in England and Wales. The landing-based regime employed by NIPS allowed individuals to freely associate within their “bubble”. Movement off-landing was restricted, except for exercise in their group, but a fairly high degree of free movement was maintained. The impact on often already fragile mental health and the possible increases in anxiety, depression as well as self-harm and suicidal ideation was a specific concern though a review found most of the research was of limited quality (Usher et al., 2020). A large qualitative study in NI reported similar findings, namely that the increased isolation had exacerbated depression, anxiety and feelings of self-harm (Hewson et al., 2020), though these had been ameliorated to some extent by the introduction of Engagement Leads and an increase of timely information. The provision of virtual visits, to replace in-person visits, was especially successful particularly when families for whom travel was an issue, enabling people in prison to see their family in the home setting. This also enabled foreign national prisoners to interact with their families, sometimes for the first time since they entered prison. This innovation was continued post pandemic in the post-pandemic era. As others have commented, these changes required a high degree of organisational collaboration especially between the NIPS management and HiP provision within the prison setting.

## Conclusion

The prison service in NI appeared to have been successful in preventing COVID-19 infections for most of the pandemic and this outcome may have been due to lower occupancy levels and greater scope to make available additional isolation arrangements and more confinement living spaces. A key difference between NI Prisons and other parts of the UK was the iterative, systematic style of interagency working that examined critically the experience of PHE and HMPPS and drew upon the lessons emanating from its membership of the Five-Nations Health and Justice group. Collectively, the group used this learning and made decisions that were responsive and dovetailed to the needs and context of the prison population in NI.

## Electronic supplementary material

Below is the link to the electronic supplementary material.

Supplementary Material 1

## Data Availability

Data availability: Department of Health. (2024). COVID-19 Dashboard Updates. Available at: https://www.health-ni.gov.uk/articles/covid-19-dashboard-updates. Crown Copyright. Date of last access: 27/09/2024. Department of Justice. (2022). Weekly Situation Reports 2021/2022. Available at: https://www.justice-ni.gov.uk/articles/weekly-situation-reports-october-2015. Crown Copyright. Date of last access: 27/09/2024.
