# Supplementary material for: The management of COVID-19 in prisons – the case of Northern Ireland
Source: Health Justice. 2025 Jul 1;13:40. doi: 10.1186/s40352-025-00350-8 (PMC12219819; doi:10.1186/s40352-025-00350-8)

**Supplementary file**

**The management of COVID-19 in prisons – the case of Northern Ireland**

**Appendix 1**

The weekly number of new COVID-19 confirmed cases among the Northern Ireland population during the study period with information on national lockdowns and public restrictions.


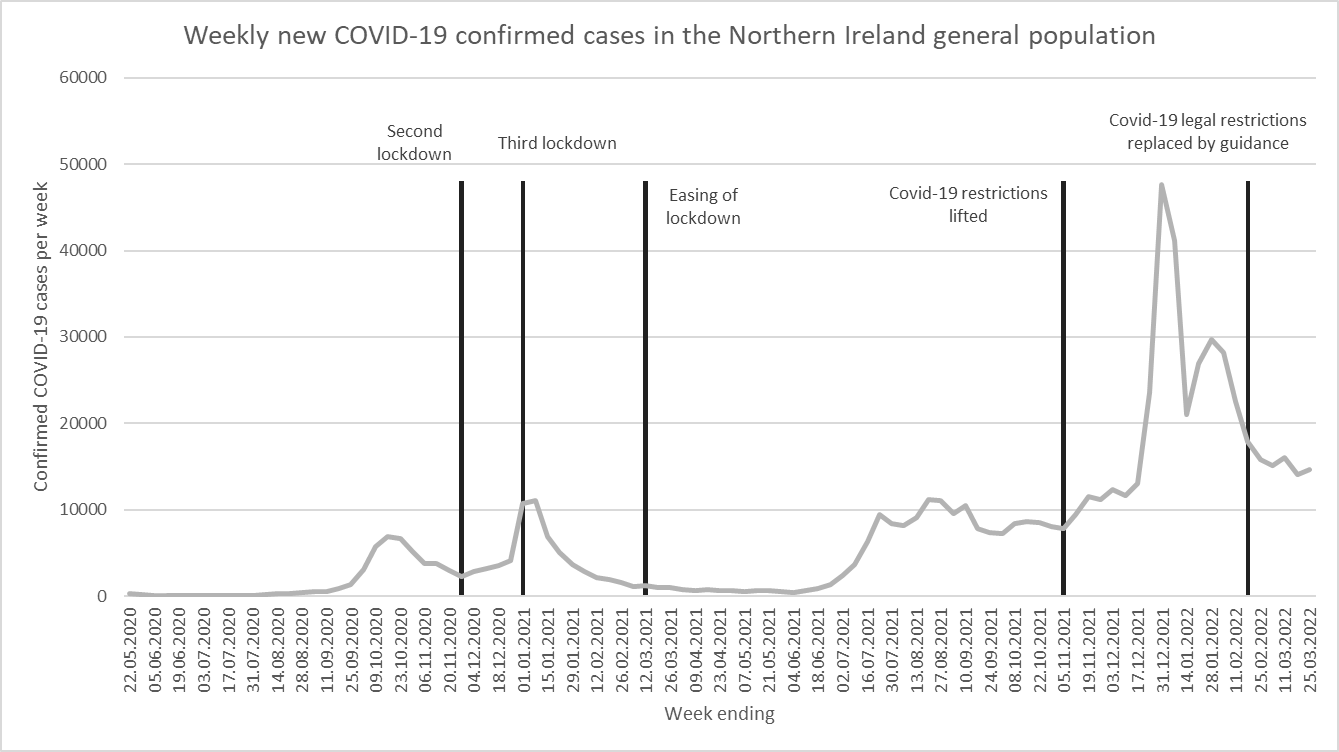

Supplement: Supplementary file 1 — Supplementary Material 1 [file 40352_2025_350_MOESM1_ESM.docx]
